# Dimethyl 4-(3-hydroxy­phen­yl)-2,6-dimethyl-1,4-dihydro­pyridine-3,5-dicarboxyl­ate

**DOI:** 10.1107/S1600536810011268

**Published:** 2010-03-31

**Authors:** K. Rajesh, V. Vijayakumar, T. Narasimhamurthy, J. Suresh, Edward R. T. Tiekink

**Affiliations:** a Organic Chemistry Division, School of Advanced Sciences, VIT University, Vellore 632 014, India; b Materials Research Centre, Indian Institute of Science, Bengaluru 560 012, India; c The Madura College (Autonomous), Madurai 625 011, India; d Department of Chemistry, University of Malaya, 50603 Kuala Lumpur, Malaysia

## Abstract

The 1,4-dihydro­pyridine ring in the title compound, C_17_H_19_NO_5_, has a flattened-boat conformation, and the benzene ring is almost orthogonal to it [dihedral angle = 82.98 (12)°]. The hydr­oxy group is disordered over two positions in a 0.780 (4):0.220 (4) ratio. In the crystal, hydrogen-bonding inter­actions of the type N_a_—H⋯O_c_ and O_h_—H⋯O_c_ (a = amine, c = carbonyl and h = hydr­oxy) link the mol­ecules into a three-dimensional network.

## Related literature

For further synthetic details, general background to this work and related structures, see: Rathore *et al.* (2009 ▶); Reddy *et al.* (2010 ▶). For ring conformations, see: Cremer & Pople, (1975 ▶).
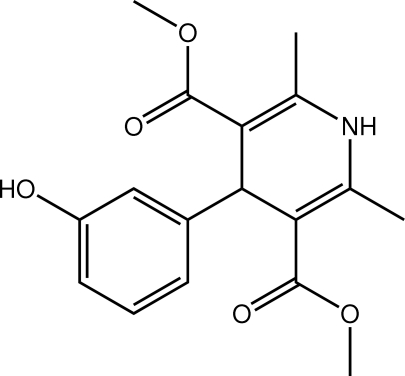


## Experimental

### 

#### Crystal data

C_17_H_19_NO_5_
*M* *_r_* = 317.33
Monoclinic, 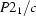

*a* = 10.4863 (7) Å
*b* = 10.4091 (7) Å
*c* = 14.8702 (11) Å
β = 99.259 (4)°
*V* = 1601.98 (19) Å^3^
*Z* = 4
Mo *K*α radiation
μ = 0.10 mm^−1^
*T* = 293 K
0.17 × 0.14 × 0.11 mm

#### Data collection

Bruker SMART APEX CCD diffractometer
Absorption correction: multi-scan (*SADABS*; Bruker, 1998 ▶) *T*_min_ = 0.646, *T*_max_ = 0.746
25465 measured reflections
2831 independent reflections
1777 reflections with *I* > 2σ(*I*)
*R*_int_ = 0.063

#### Refinement

*R*[*F* ^2^ > 2σ(*F* ^2^)] = 0.047
*wR*(*F* ^2^) = 0.140
*S* = 0.96
2831 reflections
226 parameters
6 restraints
H atoms treated by a mixture of independent and constrained refinement
Δρ_max_ = 0.20 e Å^−3^
Δρ_min_ = −0.30 e Å^−3^

### 

Data collection: *SMART* (Bruker, 2001 ▶); cell refinement: *SAINT* (Bruker, 2001 ▶); data reduction: *SAINT*; program(s) used to solve structure: *SHELXS97* (Sheldrick, 2008 ▶); program(s) used to refine structure: *SHELXL97* (Sheldrick, 2008 ▶) and *PLATON* (Spek, 2009 ▶); molecular graphics: *ORTEP-3* (Farrugia, 1997 ▶) and *DIAMOND* (Brandenburg, 2006 ▶); software used to prepare material for publication: *publCIF* (Westrip, 2010 ▶).

## Supplementary Material

Crystal structure: contains datablocks global, I. DOI: 10.1107/S1600536810011268/hb5373sup1.cif

Structure factors: contains datablocks I. DOI: 10.1107/S1600536810011268/hb5373Isup2.hkl

Additional supplementary materials:  crystallographic information; 3D view; checkCIF report

## Figures and Tables

**Table 1:** Hydrogen-bond geometry (Å, °)

*D*—H⋯*A*	*D*—H	H⋯*A*	*D*⋯*A*	*D*—H⋯*A*
N1—H1n⋯O1^i^	0.86 (1)	2.10 (1)	2.960 (3)	173 (2)
O5—H5o⋯O3^ii^	0.83 (1)	2.01 (5)	2.828 (4)	170 (6)
O5′—H5o’⋯O5^ii^	0.82 (1)	2.17 (12)	2.778 (10)	132 (1)

Symmetry codes: (i) 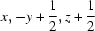; (ii) 
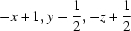
.

**Fig. 1.**
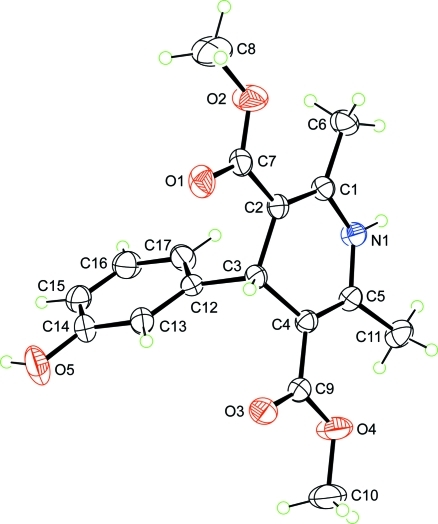
The molecular structure of (I) showing displacement ellipsoids at the 35% probability level. For reasons of clarity, only the major component of the disorder is shown.

**Fig. 2.**
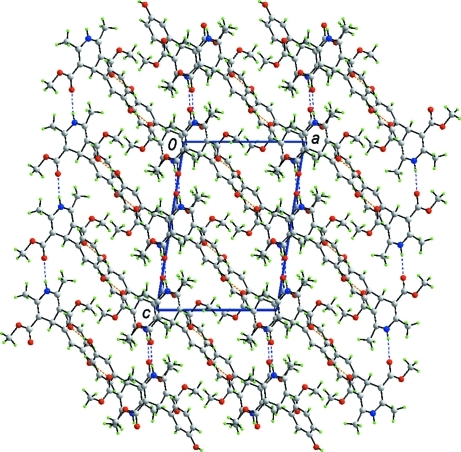
A view of the unit cell content of (I) shown in projection down the b axis. The N–H···O hydrogen bonds (blue dashed lines) link molecules into supramolecular chains along the c axis. These are connected into the three-dimensional network by O–H···O hydrogen bonds (orange dashed lines), largely obscured in the figure. Colour code: O, red; N, blue; C, grey; and H, green.

## supplementary crystallographic information

### Comment

The title compound, (I), was determined as a part of an on-going study of Hantzsch 1,4-dihydropyridines which are notable for their biological activity (Rathore *et al.*, 2009; Reddy *et al.*, 2010).

The 1,4-dihydropyridine ring in (I), Fig. 1, has a flattened-boat conformation with the N1 [0.105 (4) Å] and C3 [0.267 (4) Å] atoms lying above the least-squares plane through the C1,C2,C4, and C5 atoms r.m.s. deviation = 0.0048 Å]. The ring puckering parameters (Cremer & Pople, 1975)are Q = 0.221 (2) Å, θ = 107.0 (5) °, and φ_2_ = 357.1 (6) °. The aryl ring is orthogonal to the 1,4-dihydropyridine ring, with the dihedral angle between their respective least-squares planes being 82.98 (12) °.

The crystal structure features significant hydrogen bonding interactions, Table 1. The N_amine_–*H*···O_carbonyl_ interactions lead to chains with glide symmetry along the *c* axis. O_hydroxyl_–*H*···O_carbonyl_ hydrogen bonds exist normal to the chain resulting in a three-dimensional network, Fig. 2. As noted in the Experimental, the hydroxyl group is disordered over two positions so that the above description pertains to the major component of the structure only. The minor component of the disorder allows for the formation of O_hydroxy_–*H*···O_hydroxy_ hydrogen bonds, Table 1, to provide addiotnal cohesion to the crystal packing.

### Experimental

Dimethyl 1,4-dihydro-4-(3-hydroxyphenyl)-2,6-dimethylpyridine-3,5-dicarboxylate was prepared according to Hantzsch pyridine synthesis (Rathore *et al.*, 2009). To a mixture of 3-hydroxybenzaldehyde (1.221 g, 10 mmol), methyl acetoacetate (2.26 ml, 20 mmol) and ammonium acetate (0.771 g, 10 mmol) in ethanol (10 ml) was added and heated over water bath for about 15 minutes with shaking to ensure thorough mixing. After 15 min, the reaction mixture was kept aside for two days. The solid that separated out was filtered and washed with an ethanol/diethyl ether mixture (1:4). The purity of the crude product was checked through TLC and recrystallized from an ethanol/chloroform mixture (3:2) to yield colourless blocks of (I). Yield: 77%, m. pt. 500–501 K.

### Refinement

The C-bound H atoms were geometrically placed (C–H = 0.93–0.98 Å) and refined as riding with *U_iso_*(H) = 1.2–1.5*U_eq_*(C). The remaining H were located from a difference map and refined with O–H = 0.82±0.01 and N–H = 0.86±0.01, and with *U_iso_*(H) = n*U_eq_*(parent atom), with n = 1.5 for O and n = 1.2 for N. The 3-hydroxyl group was found to be disordered over two positions. The anisotropic displacement ellipsoids were constrained to be equal for the two hydroxyl-O atoms and the major component had a site occupancy factor = 0.780 (4).

### Crystal data

**Table:**

C_17_H_19_NO_5_	*F*(000) = 672
*M**_r_* = 317.33	*D*_x_ = 1.316 Mg m^−^^3^
Monoclinic, *P*2_1_/*c*	Mo *K*α radiation, λ = 0.71073 Å
Hall symbol: -P 2ybc	Cell parameters from 250 reflections
*a* = 10.4863 (7) Å	θ = 2.0–25.0°
*b* = 10.4091 (7) Å	µ = 0.10 mm^−^^1^
*c* = 14.8702 (11) Å	*T* = 293 K
β = 99.259 (4)°	Block, colourless
*V* = 1601.98 (19) Å^3^	0.17 × 0.14 × 0.11 mm
*Z* = 4	

### Data collection

**Table:**

Bruker SMART APEX CCD diffractometer	2831 independent reflections
Radiation source: fine-focus sealed tube	1777 reflections with *I* > 2σ(*I*)
graphite	*R*_int_ = 0.063
ω scans	θ_max_ = 25.0°, θ_min_ = 2.0°
Absorption correction: multi-scan (*SADABS*; Bruker, 1998)	*h* = −12→12
*T*_min_ = 0.646, *T*_max_ = 0.746	*k* = −12→12
25465 measured reflections	*l* = −17→17

### Refinement

**Table:**

Refinement on *F*^2^	Secondary atom site location: difference Fourier map
Least-squares matrix: full	Hydrogen site location: inferred from neighbouring sites
*R*[*F*^2^ > 2σ(*F*^2^)] = 0.047	H atoms treated by a mixture of independent and constrained refinement
*wR*(*F*^2^) = 0.140	*w* = 1/[σ^2^(*F*_o_^2^) + (0.060*P*)^2^ + 0.9343*P*] where *P* = (*F*_o_^2^ + 2*F*_c_^2^)/3
*S* = 0.96	(Δ/σ)_max_ < 0.001
2831 reflections	Δρ_max_ = 0.20 e Å^−^^3^
226 parameters	Δρ_min_ = −0.30 e Å^−^^3^
6 restraints	Extinction correction: *SHELXL97* (Sheldrick, 2008), Fc^*^=kFc[1+0.001xFc^2^λ^3^/sin(2θ)]^-1/4^
Primary atom site location: structure-invariant direct methods	Extinction coefficient: 0.0061 (15)

### Special details

**Table:**

Geometry. All s.u.'s (except the s.u. in the dihedral angle between two l.s. planes) are estimated using the full covariance matrix. The cell s.u.'s are taken into account individually in the estimation of s.u.'s in distances, angles and torsion angles; correlations between s.u.'s in cell parameters are only used when they are defined by crystal symmetry. An approximate (isotropic) treatment of cell s.u.'s is used for estimating s.u.'s involving l.s. planes.
Refinement. Refinement of *F*^2^ against ALL reflections. The weighted *R*-factor wR and goodness of fit *S* are based on *F*^2^, conventional *R*-factors *R* are based on *F*, with *F* set to zero for negative *F*^2^. The threshold expression of *F*^2^ > 2σ(*F*^2^) is used only for calculating *R*-factors(gt) etc. and is not relevant to the choice of reflections for refinement. *R*-factors based on *F*^2^ are statistically about twice as large as those based on *F*, and *R*- factors based on ALL data will be even larger.

### Fractional atomic coordinates and isotropic or equivalent isotropic displacement parameters (Å^2^)

**Table:**

	*x*	*y*	*z*	*U*_iso_*/*U*_eq_	Occ. (<1)
O1	1.00543 (18)	0.26412 (19)	0.30899 (11)	0.0533 (5)	
O2	1.1268 (2)	0.1391 (2)	0.41024 (13)	0.0691 (7)	
O3	0.69399 (19)	0.57282 (18)	0.39888 (12)	0.0550 (6)	
O4	0.6633 (2)	0.5861 (2)	0.54328 (14)	0.0695 (7)	
N1	0.9365 (2)	0.2976 (2)	0.61286 (13)	0.0415 (6)	
H1N	0.963 (2)	0.277 (2)	0.6689 (9)	0.050*	
C1	1.0040 (2)	0.2429 (2)	0.55046 (15)	0.0363 (6)	
C2	0.9694 (2)	0.2726 (2)	0.46116 (15)	0.0334 (6)	
C3	0.8480 (2)	0.3508 (2)	0.42852 (15)	0.0343 (6)	
H3	0.8676	0.4112	0.3820	0.041*	
C4	0.8074 (2)	0.4287 (2)	0.50609 (16)	0.0343 (6)	
C5	0.8478 (2)	0.3961 (2)	0.59392 (16)	0.0366 (6)	
C6	1.1099 (3)	0.1549 (3)	0.59385 (18)	0.0516 (7)	
H6A	1.0901	0.0683	0.5742	0.077*	
H6B	1.1169	0.1596	0.6589	0.077*	
H6C	1.1902	0.1805	0.5762	0.077*	
C7	1.0337 (2)	0.2271 (3)	0.38735 (17)	0.0402 (6)	
C8	1.1905 (4)	0.0913 (4)	0.3373 (2)	0.0949 (14)	
H8A	1.1286	0.0481	0.2929	0.142*	
H8B	1.2574	0.0322	0.3618	0.142*	
H8C	1.2276	0.1619	0.3090	0.142*	
C9	0.7183 (2)	0.5346 (2)	0.47692 (17)	0.0397 (6)	
C10	0.5688 (3)	0.6853 (3)	0.5194 (2)	0.0778 (11)	
H10A	0.6094	0.7591	0.4975	0.117*	
H10B	0.5318	0.7086	0.5721	0.117*	
H10C	0.5020	0.6544	0.4725	0.117*	
C11	0.8128 (3)	0.4554 (3)	0.67894 (17)	0.0533 (8)	
H11A	0.8503	0.5396	0.6873	0.080*	
H11B	0.8453	0.4027	0.7305	0.080*	
H11C	0.7206	0.4619	0.6732	0.080*	
C12	0.7382 (2)	0.2639 (2)	0.38472 (16)	0.0370 (6)	
C13	0.6625 (3)	0.2969 (3)	0.30306 (18)	0.0482 (7)	
H13	0.6791	0.3728	0.2740	0.058*	
O5	0.4875 (3)	0.2541 (3)	0.1858 (2)	0.0789 (10)	0.780 (4)
H5O	0.437 (4)	0.196 (4)	0.167 (4)	0.118*	0.780 (4)
C14	0.5613 (3)	0.2175 (3)	0.2637 (2)	0.0558 (8)	0.780 (4)
C15	0.5379 (3)	0.1037 (3)	0.3039 (2)	0.0558 (8)	0.780 (4)
H15	0.4727	0.0493	0.2765	0.067*	0.780 (4)
C16	0.6119 (3)	0.0712 (3)	0.3850 (2)	0.0583 (8)	0.780 (4)
H16	0.5955	−0.0055	0.4131	0.070*	0.780 (4)
O5'	0.5794 (11)	−0.0300 (9)	0.4212 (6)	0.0789 (10)	0.220 (4)
H5O'	0.528 (13)	−0.059 (12)	0.379 (6)	0.118*	0.220 (4)
C14'	0.5613 (3)	0.2175 (3)	0.2637 (2)	0.0558 (8)	0.220 (4)
H14'	0.5096	0.2422	0.2097	0.067*	0.220 (4)
C15'	0.5379 (3)	0.1037 (3)	0.3039 (2)	0.0558 (8)	0.220 (4)
H15'	0.4727	0.0493	0.2765	0.067*	0.220 (4)
C16'	0.6119 (3)	0.0712 (3)	0.3850 (2)	0.0583 (8)	0.220 (4)
C17	0.7108 (3)	0.1501 (3)	0.42606 (19)	0.0475 (7)	
H17	0.7591	0.1265	0.4817	0.057*	

### Atomic displacement parameters (Å^2^)

**Table:**

	*U*^11^	*U*^22^	*U*^33^	*U*^12^	*U*^13^	*U*^23^
O1	0.0573 (12)	0.0738 (14)	0.0294 (10)	−0.0004 (10)	0.0084 (9)	−0.0038 (9)
O2	0.0645 (14)	0.0967 (17)	0.0491 (12)	0.0339 (13)	0.0186 (10)	0.0002 (11)
O3	0.0681 (14)	0.0489 (12)	0.0436 (12)	0.0142 (10)	−0.0044 (9)	0.0045 (9)
O4	0.0840 (16)	0.0704 (14)	0.0561 (13)	0.0429 (13)	0.0169 (11)	0.0036 (11)
N1	0.0538 (14)	0.0458 (13)	0.0250 (11)	0.0107 (11)	0.0064 (10)	0.0065 (9)
C1	0.0368 (14)	0.0382 (14)	0.0340 (13)	−0.0006 (11)	0.0061 (11)	0.0011 (11)
C2	0.0348 (13)	0.0363 (14)	0.0290 (12)	−0.0031 (11)	0.0046 (10)	−0.0005 (10)
C3	0.0391 (14)	0.0368 (14)	0.0265 (12)	0.0012 (11)	0.0033 (10)	0.0021 (10)
C4	0.0370 (14)	0.0317 (13)	0.0340 (13)	−0.0001 (11)	0.0052 (11)	−0.0003 (10)
C5	0.0439 (15)	0.0335 (14)	0.0336 (13)	−0.0018 (12)	0.0096 (11)	−0.0001 (10)
C6	0.0528 (17)	0.0592 (18)	0.0409 (15)	0.0145 (14)	0.0020 (13)	0.0068 (13)
C7	0.0349 (14)	0.0493 (16)	0.0361 (15)	−0.0055 (13)	0.0048 (11)	−0.0050 (12)
C8	0.080 (3)	0.144 (4)	0.066 (2)	0.048 (3)	0.030 (2)	−0.015 (2)
C9	0.0432 (15)	0.0337 (14)	0.0404 (15)	0.0006 (12)	0.0010 (12)	−0.0036 (12)
C10	0.081 (2)	0.070 (2)	0.083 (2)	0.038 (2)	0.015 (2)	−0.0006 (19)
C11	0.074 (2)	0.0536 (18)	0.0348 (15)	0.0107 (15)	0.0149 (14)	−0.0015 (12)
C12	0.0357 (14)	0.0392 (15)	0.0353 (13)	0.0072 (12)	0.0032 (11)	−0.0059 (11)
C13	0.0542 (17)	0.0432 (16)	0.0429 (15)	0.0026 (13)	−0.0057 (13)	−0.0051 (12)
O5	0.089 (2)	0.0661 (19)	0.0653 (18)	−0.0201 (15)	−0.0358 (16)	0.0042 (14)
C14	0.0505 (18)	0.062 (2)	0.0482 (17)	0.0063 (15)	−0.0117 (14)	−0.0169 (15)
C15	0.0447 (17)	0.0486 (18)	0.071 (2)	−0.0035 (14)	0.0014 (15)	−0.0187 (15)
C16	0.0500 (18)	0.0465 (18)	0.078 (2)	−0.0044 (14)	0.0081 (16)	−0.0033 (15)
O5'	0.089 (2)	0.0661 (19)	0.0653 (18)	−0.0201 (15)	−0.0358 (16)	0.0042 (14)
C14'	0.0505 (18)	0.062 (2)	0.0482 (17)	0.0063 (15)	−0.0117 (14)	−0.0169 (15)
C15'	0.0447 (17)	0.0486 (18)	0.071 (2)	−0.0035 (14)	0.0014 (15)	−0.0187 (15)
C16'	0.0500 (18)	0.0465 (18)	0.078 (2)	−0.0044 (14)	0.0081 (16)	−0.0033 (15)
C17	0.0426 (16)	0.0487 (17)	0.0487 (16)	−0.0018 (13)	−0.0003 (13)	0.0022 (13)

### Geometric parameters (Å, °)

**Table:**

O1—C7	1.218 (3)	C10—H10B	0.9600
O2—C7	1.342 (3)	C10—H10C	0.9600
O2—C8	1.450 (4)	C11—H11A	0.9600
O3—C9	1.214 (3)	C11—H11B	0.9600
O4—C9	1.333 (3)	C11—H11C	0.9600
O4—C10	1.436 (3)	C12—C13	1.383 (3)
N1—C1	1.377 (3)	C12—C17	1.386 (4)
N1—C5	1.382 (3)	C13—C14'	1.397 (4)
N1—H1N	0.862 (10)	C13—C14	1.397 (4)
C1—C2	1.355 (3)	C13—H13	0.9300
C1—C6	1.502 (3)	O5—C14	1.341 (4)
C2—C7	1.456 (3)	O5—H5O	0.827 (10)
C2—C3	1.523 (3)	C14—C15	1.366 (4)
C3—C12	1.526 (3)	C15—C16	1.367 (4)
C3—C4	1.525 (3)	C15—H15	0.9300
C3—H3	0.9800	C16—C17	1.385 (4)
C4—C5	1.350 (3)	C16—H16	0.9300
C4—C9	1.465 (3)	O5'—C16'	1.255 (8)
C5—C11	1.505 (3)	O5'—H5O'	0.820 (11)
C6—H6A	0.9600	C14'—C15'	1.366 (4)
C6—H6B	0.9600	C14'—H14'	0.9300
C6—H6C	0.9600	C15'—C16'	1.367 (4)
C8—H8A	0.9600	C15'—H15'	0.9300
C8—H8B	0.9600	C16'—C17	1.385 (4)
C8—H8C	0.9600	C17—H17	0.9300
C10—H10A	0.9600		
			
C7—O2—C8	116.6 (2)	H10A—C10—H10B	109.5
C9—O4—C10	118.0 (2)	O4—C10—H10C	109.5
C1—N1—C5	124.8 (2)	H10A—C10—H10C	109.5
C1—N1—H1N	115.4 (18)	H10B—C10—H10C	109.5
C5—N1—H1N	118.9 (18)	C5—C11—H11A	109.5
C2—C1—N1	118.7 (2)	C5—C11—H11B	109.5
C2—C1—C6	128.5 (2)	H11A—C11—H11B	109.5
N1—C1—C6	112.8 (2)	C5—C11—H11C	109.5
C1—C2—C7	125.5 (2)	H11A—C11—H11C	109.5
C1—C2—C3	120.8 (2)	H11B—C11—H11C	109.5
C7—C2—C3	113.5 (2)	C13—C12—C17	118.1 (2)
C2—C3—C12	110.69 (19)	C13—C12—C3	121.0 (2)
C2—C3—C4	111.33 (18)	C17—C12—C3	120.9 (2)
C12—C3—C4	110.8 (2)	C12—C13—C14'	120.7 (3)
C2—C3—H3	108.0	C12—C13—C14	120.7 (3)
C12—C3—H3	108.0	C12—C13—H13	119.6
C4—C3—H3	108.0	C14'—C13—H13	119.6
C5—C4—C9	124.2 (2)	C14—C13—H13	119.6
C5—C4—C3	121.0 (2)	C14—O5—H5O	109 (4)
C9—C4—C3	114.7 (2)	O5—C14—C15	120.4 (3)
C4—C5—N1	118.7 (2)	O5—C14—C13	119.2 (3)
C4—C5—C11	128.9 (2)	C15—C14—C13	120.5 (3)
N1—C5—C11	112.4 (2)	C16—C15—C14	118.9 (3)
C1—C6—H6A	109.5	C16—C15—H15	120.5
C1—C6—H6B	109.5	C14—C15—H15	120.5
H6A—C6—H6B	109.5	C15—C16—C17	121.4 (3)
C1—C6—H6C	109.5	C15—C16—H16	119.3
H6A—C6—H6C	109.5	C17—C16—H16	119.3
H6B—C6—H6C	109.5	C16'—O5'—H5O'	100 (8)
O1—C7—O2	120.9 (2)	C15'—C14'—C13	120.5 (3)
O1—C7—C2	123.2 (2)	C15'—C14'—H14'	119.8
O2—C7—C2	115.9 (2)	C13—C14'—H14'	119.8
O2—C8—H8A	109.5	C16'—C15'—C14'	118.9 (3)
O2—C8—H8B	109.5	C16'—C15'—H15'	120.5
H8A—C8—H8B	109.5	C14'—C15'—H15'	120.5
O2—C8—H8C	109.5	O5'—C16'—C15'	115.5 (5)
H8A—C8—H8C	109.5	O5'—C16'—C17	123.0 (5)
H8B—C8—H8C	109.5	C15'—C16'—C17	121.4 (3)
O3—C9—O4	121.8 (2)	C16'—C17—C12	120.3 (3)
O3—C9—C4	123.7 (2)	C16—C17—C12	120.3 (3)
O4—C9—C4	114.5 (2)	C16'—C17—H17	119.8
O4—C10—H10A	109.5	C16—C17—H17	119.8
O4—C10—H10B	109.5	C12—C17—H17	119.8
			
C5—N1—C1—C2	−10.3 (4)	C5—C4—C9—O3	171.2 (3)
C5—N1—C1—C6	169.9 (2)	C3—C4—C9—O3	−11.8 (4)
N1—C1—C2—C7	177.5 (2)	C5—C4—C9—O4	−9.6 (4)
C6—C1—C2—C7	−2.6 (4)	C3—C4—C9—O4	167.4 (2)
N1—C1—C2—C3	−7.7 (4)	C2—C3—C12—C13	−134.6 (2)
C6—C1—C2—C3	172.1 (2)	C4—C3—C12—C13	101.4 (3)
C1—C2—C3—C12	−102.0 (3)	C2—C3—C12—C17	45.9 (3)
C7—C2—C3—C12	73.3 (3)	C4—C3—C12—C17	−78.0 (3)
C1—C2—C3—C4	21.7 (3)	C17—C12—C13—C14'	0.1 (4)
C7—C2—C3—C4	−163.0 (2)	C3—C12—C13—C14'	−179.4 (2)
C2—C3—C4—C5	−20.8 (3)	C17—C12—C13—C14	0.1 (4)
C12—C3—C4—C5	102.9 (3)	C3—C12—C13—C14	−179.4 (2)
C2—C3—C4—C9	162.1 (2)	C12—C13—C14—O5	178.1 (3)
C12—C3—C4—C9	−74.3 (3)	C12—C13—C14—C15	−2.0 (4)
C9—C4—C5—N1	−177.2 (2)	O5—C14—C15—C16	−177.6 (3)
C3—C4—C5—N1	5.9 (4)	C13—C14—C15—C16	2.4 (4)
C9—C4—C5—C11	0.6 (4)	C14—C15—C16—C17	−1.0 (5)
C3—C4—C5—C11	−176.2 (3)	C12—C13—C14'—C15'	−2.0 (4)
C1—N1—C5—C4	11.2 (4)	C13—C14'—C15'—C16'	2.4 (4)
C1—N1—C5—C11	−167.0 (2)	C14'—C15'—C16'—O5'	175.2 (7)
C8—O2—C7—O1	−0.2 (4)	C14'—C15'—C16'—C17	−1.0 (5)
C8—O2—C7—C2	179.1 (3)	O5'—C16'—C17—C12	−176.8 (7)
C1—C2—C7—O1	−174.1 (2)	C15'—C16'—C17—C12	−0.9 (4)
C3—C2—C7—O1	10.8 (3)	C15—C16—C17—C12	−0.9 (4)
C1—C2—C7—O2	6.6 (4)	C13—C12—C17—C16'	1.3 (4)
C3—C2—C7—O2	−168.4 (2)	C3—C12—C17—C16'	−179.2 (2)
C10—O4—C9—O3	2.8 (4)	C13—C12—C17—C16	1.3 (4)
C10—O4—C9—C4	−176.3 (2)	C3—C12—C17—C16	−179.2 (2)

### Hydrogen-bond geometry (Å, °)

**Table:**

*D*—H···*A*	*D*—H	H···*A*	*D*···*A*	*D*—H···*A*
N1—H1n···O1^i^	0.862 (10)	2.103 (14)	2.960 (3)	172.8 (19)
O5—H5o···O3^ii^	0.827 (10)	2.01 (5)	2.828 (4)	170 (6)
O5'—H5o'···O5^ii^	0.820 (11)	2.17 (12)	2.778 (10)	132 (1)

Symmetry codes: (i) *x*, −*y*+1/2, *z*+1/2; (ii) −*x*+1, *y*−1/2, −*z*+1/2.
